# Guide dogs' navigation after a single journey: A descriptive study of path reproduction, homing, shortcut and detour

**DOI:** 10.1371/journal.pone.0219816

**Published:** 2019-07-16

**Authors:** Florence Gaunet, Sandie Besse

**Affiliations:** 1 Laboratoire de Psychologie Cognitive, Aix-Marseille University - CNRS, Fédération 3C Marseille, France; 2 Les chiens guides d’aveugles de Provence Côte d’Azur Corse, Lançon de Provence, France; University of Lethbridge, CANADA

## Abstract

Guide dogs are working dogs that follow the verbal instructions of owners with severe visual impairments, leading them through the environment and toward goals such as a subway entrance (“Find the subway” instruction). During this process, guide dogs incidentally familiarize themselves with their environment. As such, they provide a unique animal model for studying wayfinding abilities in the canine species. In the present descriptive study, 23 skilled guide dogs travelled along a path once and were subsequently tested in a navigation task, with a blindfolded guide dog instructor as the handler. Dogs had difficulty reproducing the path (only 30.43% of the dogs succeeded) and returning (homing) along the previously travelled path (43.47% of the dogs succeeded). However, 80% of them successfully took a shortcut, and 86.95% a detour. This is the first description of the wayfinding abilities of dogs after a single discrete exploration of the path (incidental learning) in systematic experimental conditions. Errors, initiatives and success rates showed that dogs were able to keep track of the goal if the path was short, but errors increased with longer paths, suggesting segmented integration of path characteristics process, as demonstrated in human**s**. Additionally, errors on homing and detouring, both vital wayfinding tasks, were correlated, suggesting an effect of experience. Initiatives taken by the dogs further suggest flexibility of the spatial representation elaborated. Interestingly, we also found that homing was the only task to benefit from severe visual disability and regular exposure to new journeys, suggesting that these two factors influence the most important wayfinding task. This study therefore highlights qualitative and quantitative wayfinding abilities in the dog species, as well as the factors that account for them, after a single path exploration accompanied by natural ongoing motivation. In the wake of the discovery that dogs are sensitive to the magnetic field, our results provide the basis for developing systematic wayfinding tests for guide dogs.

## Introduction

We are not aware of any previous study that has focused on navigational abilities per se outdoors during usual travelling situations, either in guide dogs or in pet dogs. Given that wayfinding is a major cognitive ability of Canids, and dogs have recently been shown to be sensitive to the magnetic field [1,2], it is important to initiate research on navigation and its foundations, and guide dogs are the best candidates for this because of their expertise in the domain.

The basic abilities of guide dogs were described by our colleagues [3] in 1997. These service dogs [4] provide mobility and orientation for individuals with severe visual impairments (Mobility & Orientation [5,6]). Mobility refers to find important items in the environment. The dog signals these items to its owner by stopping at the target: some without any verbal instruction from the owner (e.g. stairs, curbs at pedestrian crosswalks, sidewalks); others solely after a verbal instruction (e.g. doorways, benches, bus stops, subway entrances). In the latter case, where the instructions are “Find the metro/chair”, the verb “find*”* have been previously associated with the “going to” action and a referent/object/place. Owners may subsequently teach their dogs new items. This particular learning of using “Find [item]”, where the item is known, is the very reason why guide dogs are such good candidates for studying wayfinding in the canine species. Guide dogs identify and avoid obstacles while walking (e.g. tables and chairs on sidewalks, holes in the ground resulting from insufficient roadwork safety) without any instruction: they integrate their owner’s body schema into their own and are taught to intelligently disobey (e.g. change direction, refuse to go on) if they encounter obstacles. In the meanwhile, guide dogs mobilize their orientation (i.e. wayfinding or navigational) abilities. They use directional verbal instructions (“Go on”, “Straight”, “Right”, “Left”, “Stop”) to follow unfamiliar paths their owners prepared earlier (e.g. use of written instructions or GPS), where different surfaces (sidewalks and pedestrian crossings) constrain the dog’s walking direction. The dogs learn the paths and the environment they go through, just as any mammal would [7–9], for incidental spatial learning, see also [10]. Whereas the dogs need to be instructed to reach a specific goal (e.g. “Find the baker, baker”, “Find Mum, Mum”, “Find home, home”), once they have become familiar with a path or environment, verbal directional instructions become almost unnecessary, although encouragement and the “Go on” instruction after stops are still needed. As the dogs’ orientation ability develops, their spatial knowledge can be used more flexibly (going somewhere by bus and returning on foot, detouring, shortcutting). It is important to note that once a guide dog has been acquired, it accompanies its owner on all his/her journeys. The frequency and type of journey therefore depend on that person’s general orientation skills, habits and needs [11], as well as on the dogs’ individual abilities.

In familiar environments, individuals perform four tasks: they reproduce formerly travelled paths, travel to places and return via the same route (round trip/homing), and perform shortcuts or detours around blocks of houses or buildings when an obstacle is present [7, 12]. These abilities have been observed in many animal species [7, 8]. Whereas the first task can be defined as the exact “reproduction*”* of a previously performed activity, “homing” requires the processing of a previously travelled path from a new vantage point, and “shortcutting” involves keeping track of the direction and distance of a goal while moving ever closer to it. “Detouring” relies on exactly the same process, except that the individual moves ever further from the goal.

Spatial cognition is an essential cognitive function for survival in almost all species: it enables animals to navigate through complex environments [8–9]. Animals use a number of cues and mechanisms for navigation [13–15]. These include both intrinsic cues provided by the animal’s own movements, and extrinsic cues emanating from the animal’s environment. Intrinsic cues are used by numerous insects and vertebrates to take a direct path home after following a route to some distant point (path integration). Extrinsic environmental cues, on the other hand, allow animals to navigate through spatial environments by using geometric relationships between objects [16], landmarks and beacons [17], and by computing distance and direction vectors using multiple landmarks [18]. Dogs have been shown to be capable of resolving invisible displacement tasks [19–21], and for more complex tasks, see [22, 23]. [24] showed that dogs use path integration. [25] also found that dogs encoded target locations, using both the landmarks and the global cues provided by the testing room. [26] suggested that dogs use both egocentric cues (turning left or right) and allocentric cues (relative position of objects in their surrounding space) in a hierarchical fashion. When dogs can take a linear path between their position and a target location, they maintain a vector containing information about the target’s distance and direction information even when a barrier is placed between them and the target. When dogs search for an object that has disappeared or been visibly displaced, their preferred strategy is to use egocentric cues to find it, such as a linear path or dead reckoning. If these cues are unavailable, however, dogs use allocentric cues, such as the position of the target relative to landmarks and global cues [27–30]. However, although considerable research has been carried out on spatial memory in laboratory settings, no work has so far examined dogs’ wayfinding ability per se.

In the present study, we developed and administered four tests to probe the four key navigational tasks in outdoors usual setting. After visiting a path once, guide dogs (all trained at one particular guide dog school) were tested on each navigational ability. We measured the numbers and percentages of dogs that succeeded, the number of errors, the type of errors and initiatives (i.e. unexpected but appropriate choices of direction, given their training), and the durations of the paths in the memorization and evaluation phases. Our aim was to identify the level of navigational ability a guide dog can reach in each task (with descriptive statistics). Given the lack of data on this topic, we could not form any hypotheses. However, as trainers, and here S.B., know that guide dogs and their owners usually require at least three or four visits to become familiar with a 300-400 meter path (see S1 Text), we did not expect the dogs to become skilled in any of the tasks. The nature of their errors and initiatives would shed light on early (i.e. after a single path experience) memorization processes and how they are related to path learning in humans (i.e. use of ego- vs. exocentric frames of reference). We also analyzed whether the different tasks were related in terms of errors and the numbers and percentages of dogs that succeeded. We expected to find links between path reproduction and homing (simple memory of places) and between shortcutting and detouring (manipulation of mental representations) [9]. Homing and detouring might also be linked, as these are the tasks most frequently performed by guide dogs in cities, and would therefore reflect an effect of experience. Finally, the study explored relationships between the number of dogs that succeeded and their individual characteristics.

## Material & methods

The guide dogs belong to the school ‘Ecole de Chien Guide du Midi’ and are loaned to visually impaired persons. In the context of a checking/control visit, the dogs participated to the observational experiment on their usual activity: navigation. The study was conducted in accordance to the legal requirements of France (where it was carried out), and the institutional guidelines of the Aix-Marseille Université, France. Co-author S.B. who appears as the experimenter in the S1 Video has given written informed consent (as outlined in PLOS consent form) to appear in the video.

### 2.1 Participants

The 23 guide dogs were already expert working dogs that had all been trained earlier at the Ecole de Chien Guide du Midi (Aix en Provence, France). Table 1 shows the characteristics of both the dogs and their owners. The criteria for selecting the dogs were 1) at least 1 year of cohabitation with its owner, and 2) less than 10 years old, and far from retirement. Availability was the last criterion.

**Table 1:** Characteristics of the dogs.

Characteristics	Mean (*M*), standard error (*SE*), or frequency
**Age of dogs**	*M* = 5.4 years, *SE* = 1.5
**Length of cohabitation between dog and owner**	*M* = 2.7 years, *SE* = 1.5
**Breeds**	1 German shepherd, 18 labradors, 4 flatcoats
**Sex of dogs**	12 males, 11 females
**Instructors involved in previous training**	14 by S, 8 by C, and 1 by J (S, C and J were the school’s three instructors)
**Degree of owner’s visual impairment**	6 partially sighted, 4 with light perception, 13 totally blind
**Types of paths usually travelled by the owner and his/her dog at the time of testing, according to the instructor team and each owner. Total: 23 dyads.**	“Regular” (only regular paths were travelled): 8 (according to instructor team) and 10 (according to owner) dyads “Regular + few unknown” (regular paths were mainly travelled, and unknown paths were travelled once or twice a month): 10 (according to instructor team) and 8 (according to owner) dyads “Regular + many unknown” (regular paths were travelled, unknown paths were travelled at least once a week in a familiar city, and unknown cities were visited at least once a year): 5 (according to instructor team) and 5 (according to owner) dyads

### 2.2 Paths & material

**Fig 1:**
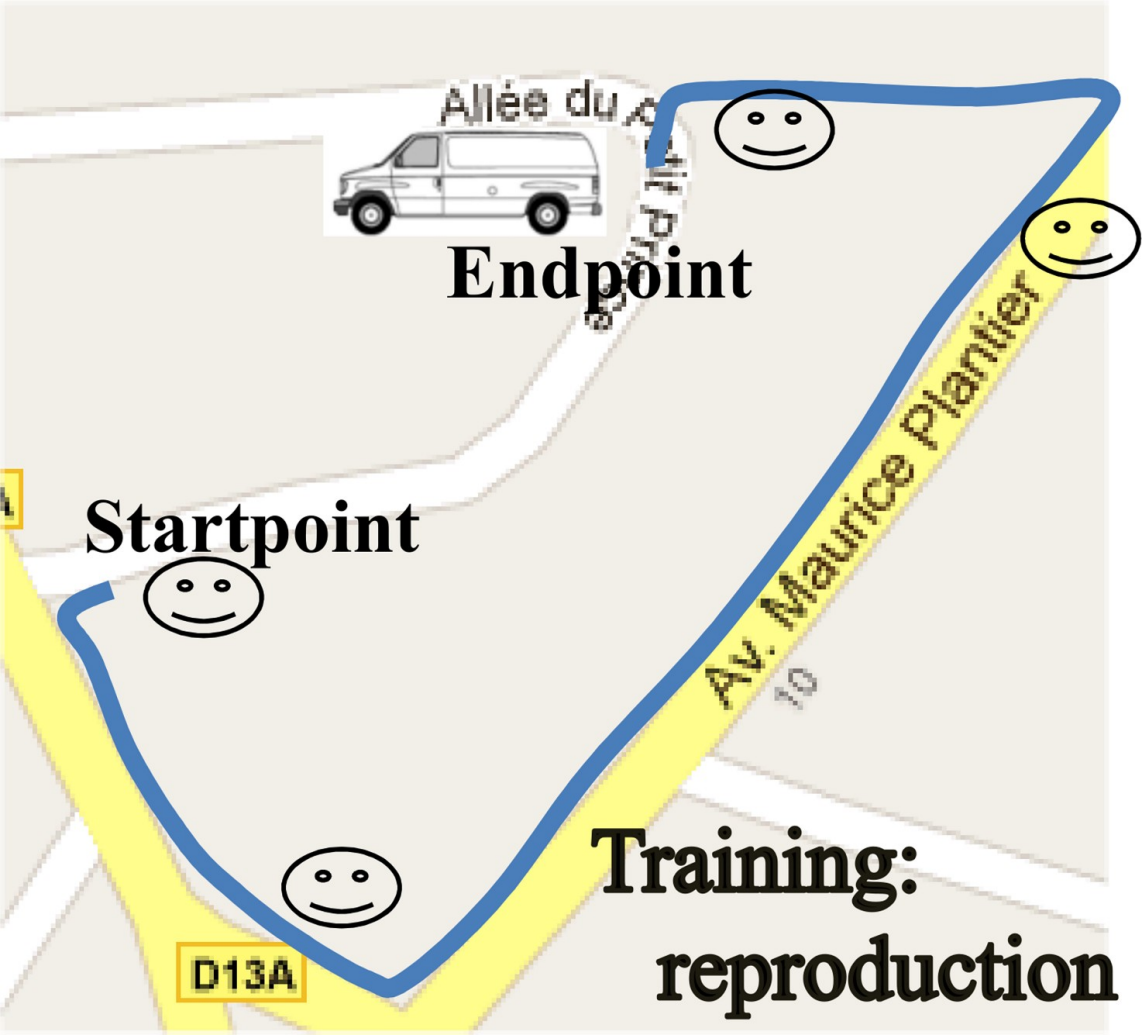
Path for the Selection and training phase: The solid line indicates the path followed during the Memorization phase, with the start and endpoints. The same path had to be reproduced in the Evaluation phase. Smileys show where the dogs were told to find the van.

**Fig 2:**
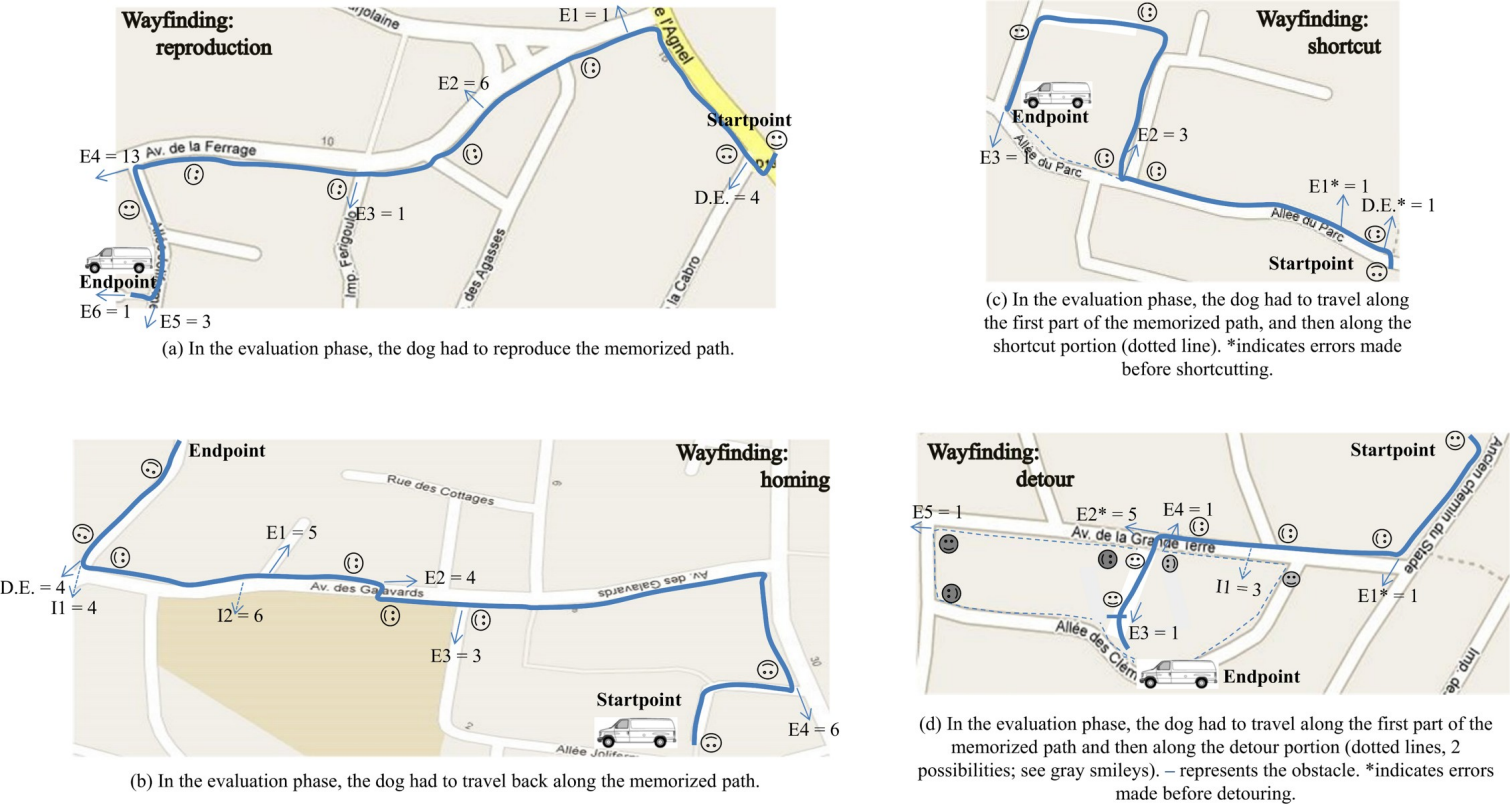
Paths for the wayfinding phase: the solid blue lines indicate the paths followed during the Memorization phase for the (a) reproduction, (b) return/homing, (c) shortcut and (d) detour tasks, with the start and endpoints. Smileys show where the dogs were told to find the van during the Evaluation phase. The figures also show the locations of the different types of errors during the Evaluation phase (D.E. for Departure Error, and E_X_ for Error number x; see solid arrows) and the total numbers of errors made, as well as initiatives (I_X_ for Initiatives number x; see dotted arrows) and their total numbers.

We designated five different paths–none known by the dogs–in a residential area. The paths had to be incidentally memorized by each dog, based on one journey along the path. One path was used for dog selection and training purposes, where we ensured that the dogs could learn the name of a target (“van”) and go back to that target after a walk when it was named, which is what happens in everyday life and when dogs and their owners take familiar paths (cf. see S1 Text). For details of this phase, see Section 2.3.1. “Selection & training phase”. The four others served as a testing ground for the four wayfinding tasks (see Section 2.3.2. “Wayfinding phase”). The dogs always wore their harness, indicating to them that they were at work. The path the dogs had to memorize for the Selection and training phase was 208 m long (see Fig 1). The four paths they had to memorize for the wayfinding tasks are depicted in Fig 2. These measured 298 m for the reproduction task, 382 m for the homing task, 258 m for the shortcut, and 172 m for the detour. All paths are scaled identically in the figures.

A camera with a wide-angle lens was used to record the dogs’ behaviors. A van was used to transport the dog, the videographer (FG) and the dog handler (SB) between the start and end points of the paths that were to be travelled. The dog could not see outside.

### 2.3 Procedure

The dogs were individually tested. Both the Selection and training phase and the Wayfinding phase were subdivided into a memorization and an evaluation phase.

#### 2.3.1. Selection & training phase

First, each dog was taught that the goal it had to reach (i.e. the van) was associated with the word *van*, so that it would lead the handler back to this specific goal after a walk, upon receiving the instruction “Find the van”. To this end, while standing near the van, the handler provided the dog with food while indicating and touching/tapping the van and repeating the word *van*. The dog received four treats, one for each repetition of the word. This is a classic method used by instructors to trigger the association between a word and an object (e.g. chair).

**Fig 3:**
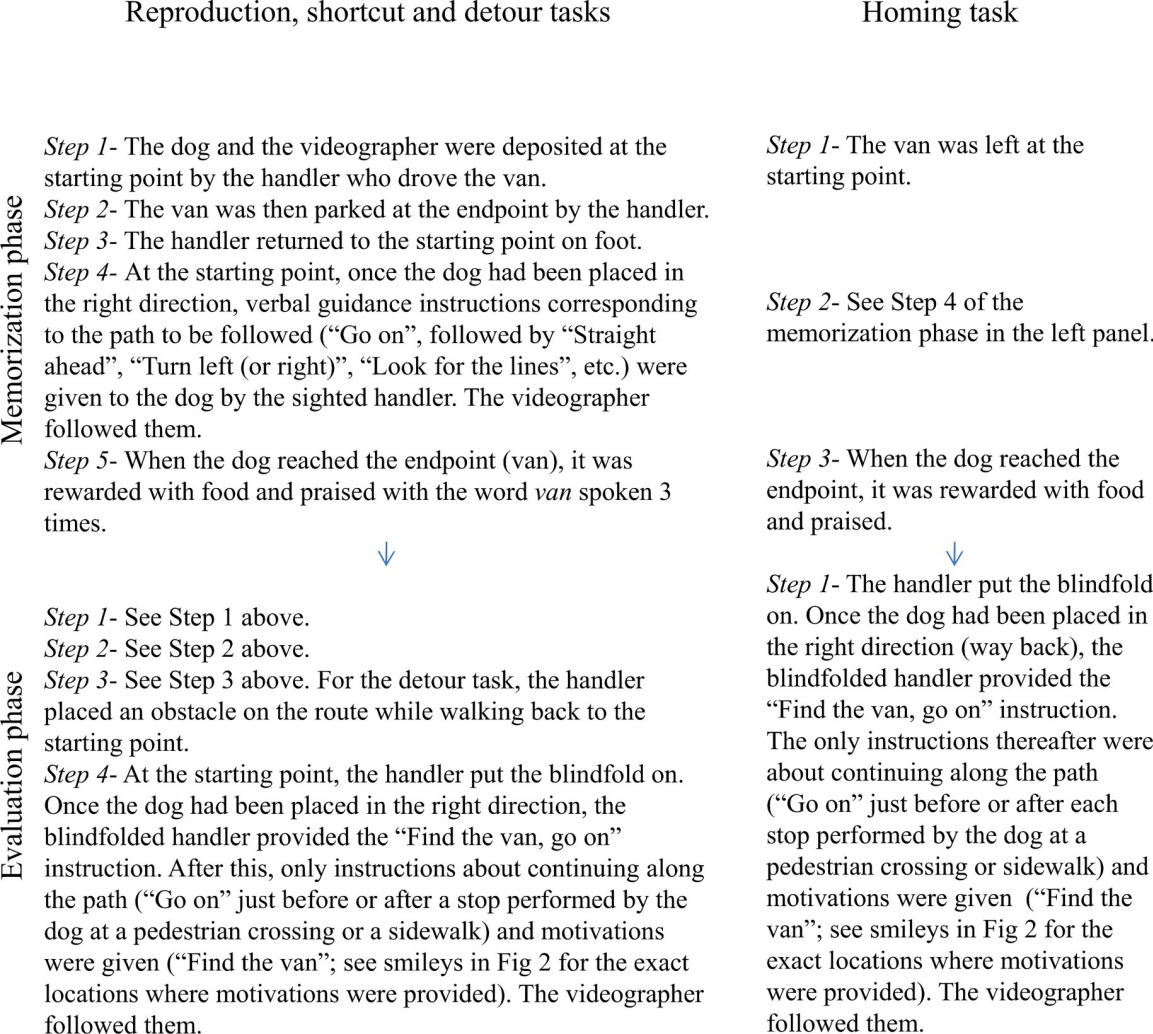
Procedure followed for the Selection and training phase and the wayfinding phase.

The second step focused on training the dog for the tasks it would have to perform during the wayfinding phase: encouraging the dog to memorize a path from starting point to endpoint, and assessing its wayfinding ability. We chose the reproduction task for this purpose, illustrated in Fig 3 (left panel). There was a 7-minute interval between the memorization and evaluation phases; this duration was chosen because it happened to be the longest duration between the end of the memorization of a path and the evaluation phase; for the Reproduction task as a matter of facts (see below). If the dog successfully completed the Selection and training phase (i.e. returned to the van without a single error), it could graduate to the Wayfinding phase (only one dog failed, and did not participate in the study).

#### 2.3.2. Wayfinding phase

Each path served one of four discrete tasks (see Fig 2):

Reproduction task. The dog was led along the path it had to memorize. Next, for the evaluation, the dog was placed back at the starting point and had to reproduce it exactly (298 m). If the dog failed to reproduce the path, we stopped it as soon as it took a wrong direction, placed it at the location where it had taken the wrong turn, and corrected its orientation. Next, we gave the “Find the van, go on” instruction.

Homing task. The dog was led along the path it had to memorize. For the evaluation, it had to walk all the way back (382 m). If the dog failed to return to the starting place, we stopped the dog as soon as it took a wrong direction, and applied the same procedure as for the reproduction task. If the dog crossed the Avenue des Galavards before the pedestrian crossing it had traversed during the memorization phase, but kept moving toward the goal, we did not interrupt its walk (see definition of initiative below). In pretests with nine dogs, some of the dogs continued along the sidewalk instead of crossing the Avenue des Galavards to the right, but they all lost their way at the end of this avenue (i.e. they turned left instead of crossing the Avenue des Galavards). For this reason, we interrupted the dog’s walk if it took that direction.

Shortcut task. The dog was led along the path it had to memorize. Next, for the evaluation, we placed it back at the starting point. In the course of the walk, it had to find another possible path to perform a shortcut to reach the endpoint. If the dog failed to reproduce the path until it reached a possible shortcut, or failed to continue in the direction of the endpoint while on either possible route toward it (shortcut or memorized path), we applied the same procedure as in the first two tasks.

Detour task. The dog was led along the path it had to memorize. Next, for the evaluation, we placed it back at the starting point, but this time placed an obstacle in the memorized route (two barrier tapes), forcing the dog to find another path. There were two possible detours: 302 m to the south and 284 m to the north. If the dog failed to reproduce the path leading to the obstacle or did not continue in the direction of the endpoint while on either detour, we applied the same procedure as before. Pretesting with nine dogs had revealed that some dogs crossed in front the Avenue de la Grande Terre at the very beginning, instead of continuing along the sidewalk. Where this occurred, we interrupted the dog’s walk.

The goals were never visible from the departure point. In all four tasks, there was a 7-minute interval between the end of the memorization phase and the beginning of the evaluation phase. The order of task presentation was randomized across the dogs. The whole experiment lasted between 1 hour and 45 minutes and 2 hours and 15 minutes, depending on the order of the tasks, as this altered the amount of time needed to drive from area to area. The interval between each task version was 15 min. in average; it was the maximal duration to go from site to another.

FG walked behind the dyad and filmed a 1.5 m area around the dyad on all four paths. She used a map of each path and indicated the precise location where the handler had to give the dog a motivational instruction (“Here”). With this instruction, the handler motivated the dog with a brief reminder of the intended goal, as is usually done for guide dogs, in a positive intonation (see Fig 3 for motivation locations). To determine the optimum number and location of the motivational instructions intended to maintain the dog’s engagement in the evaluation phase, we pretested eight of the nine earlier dogs used for the pre-tests.

The handler carried a blindfold with her for the evaluation phase. She also brought small dog treats so that the dogs would continue to associate the van with a reward: the dog was rewarded when it reached the van, in both the Selection and training (memorization) and Wayfinding phases (memorization and evaluation phases).

## Behavioral recording and data analyses

Any locations and durations of behavior (see Section 3.1.) that did not correspond to the expected (correct) responses (i.e. errors) were removed from the videos by one of the judges (FG, see S2 Text), as in [31, 32]. We computed five variables (percentages of dogs that successfully completed the tasks, number of errors, types of errors, types of initiatives, and durations of the memorized and evaluated paths) for each task and for all the dogs taken together. Descriptive statistics are provided for each task (see Section 3.1.). We also calculated the relationships between the tasks in terms of errors and the percentages of dogs that succeeded (see Section 3.2.), and between the dogs that succeeded and their individual characteristics (see Section 3.3.).

### 3.1 Percentages of dogs that succeeded, number of errors, types of errors, types of initiatives, and durations of the memorized and evaluated paths

The rules for counting errors on each task are set out in Table 2. We recorded both the error and the time it occurred. The error ended when the dog received the instruction “Find the van, go on” (i.e. after it had been repositioned and correctly oriented). Based on our recording of the errors, we were able to compute dogs that succeed and the percentage of dogs that successfully completed each path/task and the total number of errors for the group.

**Table 2:** Behaviors accounting for an error.

Task	An error was counted when:
**Reproduction**	The dog did not follow the path performed during the memorization phase.
**Homing**	The dog did not follow the route to return to the starting point; an exception was when the dog crossed the Avenue des Galavards before the pedestrian crosswalk used during the memorization phase, but continued to head toward the goal.
**Shortcut**	The dog did not reproduce the path as far as the possible shortcut, or did not follow the direction of the endpoint while on either possible route toward it (i.e. shortcut or memorized path).
**Detour**	The dog did not reproduce the path as far as the obstacle or did not head toward the endpoint while on either of the detour paths.

In principle, there was a large number of possible errors and initiatives. Table 3 shows the types of errors we actually observed for each task, and Table 4 shows the initiatives we actually observed for each task (see Fig 2 for the locations and numbers of errors and initiatives we observed).

**Table 3:** Types of errors observed for each task. For the shortcut and detour tasks, errors that occurred before the location of shortcutting or detouring are marked with an *; see also Fig 2.

Task	Departure error (D.E.)	Error 1 (E1)	Error 2 (E2)	Error 3 (E3)	Error 4 (E4)	Error 5 (E5)	Error 6 (E6)
**Reproduction**	The dog entered the street almost opposite after crossing.	The dog crossed the street to the right (no pedestrian crossing).	The dog crossed the street to the right (pedestrian crossing).	The dog entered the street on the left.	The dog crossed the street in front.	The dog continued straight ahead.	The dog passed the van, continuing straight ahead.
**Homing**	The dog continued along the sidewalk.	The dog entered the street on the left.	The dog continued along the sidewalk.	The dog entered the street on the right.	The dog crossed the street in front.	/	/
**Shortcut**	The dog entered an open parking area after crossing.*	The dog entered the street on the right.*	The dog entered the street on the right (i.e. reproducing the memorized path).	The dog crossed the street to the left before seeing the van (no pedestrian crossing).	/	/	/
**Detour**	/	The dog crossed the street to the left.*	The dog continued along the sidewalk.*	The dog could not avoid the obstacle.	The dog crossed back at the pedestrian crossing.	The dog crossed the street in front (no pedestrian crossing).	/

**Table 4:** Initiatives taken during each task.

Task	Initiative 1 (I1)	Initiative 2 (I2)
**Reproduction**	/	/
**Homing**	The dog crossed Avenue des Galavards at the very first possible location (no pedestrian crossing).	The dog crossed Avenue des Galavards 60 m later, at the very first possible location (pedestrian crossing).
**Shortcut**	/	/
**Detour**	The dog crossed Avenue Grande Terre to the left, 20 m before the pedestrian crossing (no pedestrian crossing).	/

We computed the duration of each path during the memorization phase, and then during the evaluation phase, where we subtracted the summed duration of the errors from the total duration of the path evaluated.

Descriptive statistics for the percentages of dogs that succeeded, the total number of errors, the types of errors and initiatives, and the durations of the memorized and evaluated paths for each task are provided in the Results section for each task. For each task, we compared the durations of the memorized and evaluated paths (Wilcoxon signed-rank test), and both were submitted to Spearman correlations. Additionally, as some errors in the shortcut and detour tasks did not concern shortcutting and detouring abilities per se, we followed up with an additional step where the dogs concerned were removed from the computation of the errors.

### 3.2 Relationships between the tasks in terms of errors and the percentages of dogs that succeeded

We ran the Spearman correlation test to analyze whether there was a relationship between each pair of tasks, and between the two tasks involving simple memory of places (reproduction and homing scores pooled) and the two tasks requiring the manipulation of mental representations (shortcutting and detouring scores pooled), for errors and percentages of dogs that succeeded.

### 3.3 Relationships between dogs that succeeded and individual characteristics

We focused solely on dogs that succeeded vs. failed, as this variable directly reflected a required ability in guide dogs. We used the Spearman correlation test to determine whether the percentages of dogs that succeeded on each task were linked to the two quantitative individual characteristics (dog’s age and amount of time with owner). For the four qualitative individual characteristics, we used chi-square analyses to evaluate the relationship with the percentage of dogs that succeeded. We did not study instructors and breeds because of the small number of items for each factor (see Table 1). For 2 × 2 contingency tables, we applied Yates’s correction for continuity [33] when the smallest expected cell frequency fell to between 5 and l0. For the types of paths performed daily, we ended up using two categories rather than three, as the owners’ comments suggested that there was little difference between practicing regular paths versus regular + a few unknown paths. We therefore pooled regular paths and regular + few unknown paths and compared them with regular + many unknown paths.

Finally, we analyzed whether our estimations of the paths travelled by the dyads in daily life differed, and whether there were any correlations between instructors and owners (Wilcoxon signed-rank test and Spearman correlation), to control for any divergence between the two types of estimations.

## Results

**Fig 4:**
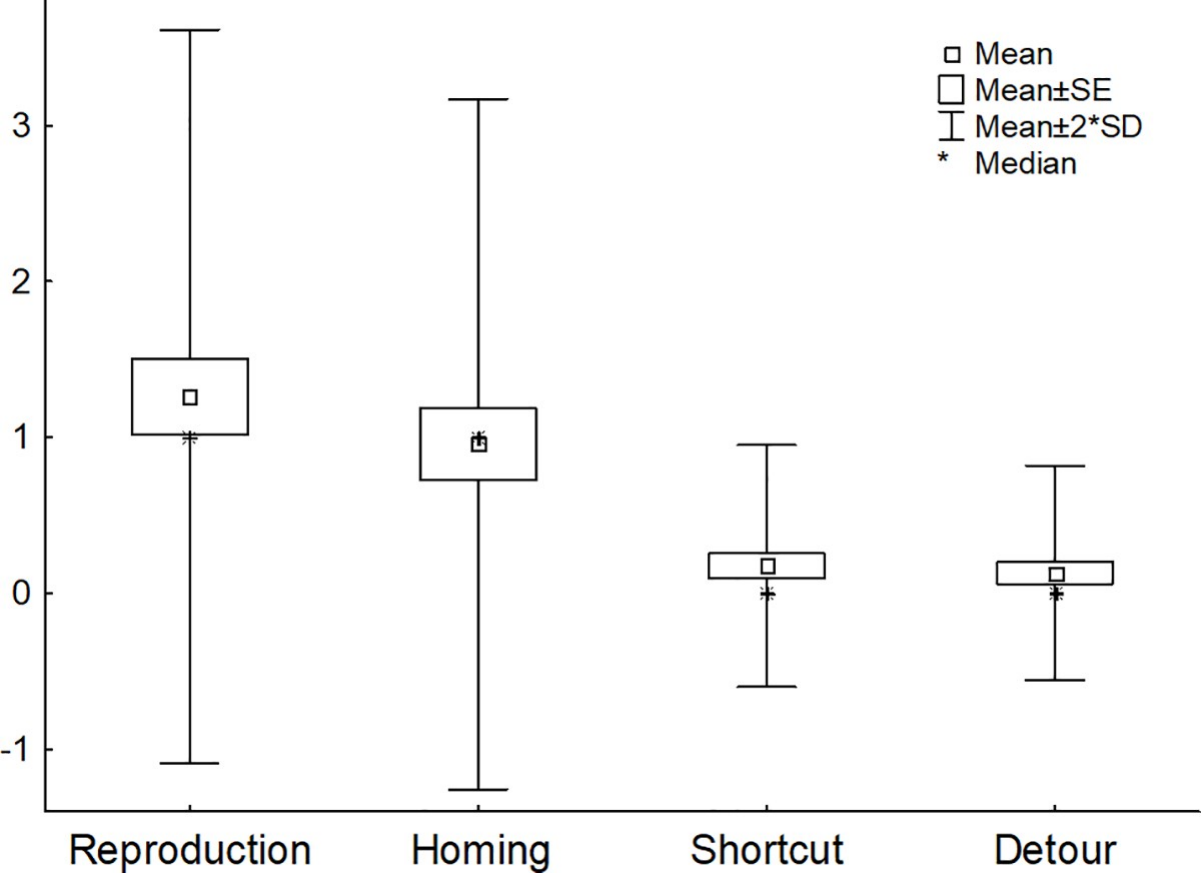
Means of the number of errors for the reproduction, homing, shortcut and detour tasks.

Fig 4 shows the means of the number of errors according to the tasks.

### 4.1 Percentages of dogs that succeeded, number of errors, types of errors and initiatives, and durations of the memorized and evaluated paths

#### Reproduction

Seven (30.43%) of the dogs successfully completed the task, while the other 16 dogs made a total of 29 errors (see Fig 2). Five of these 29 errors seemed to indicate that the dogs were keeping track of the van’s position (i.e. D.E. and E3). They behaved as though they had not memorized where exactly to turn (i.e. as though they were anticipating a turn) or were trying to take a shortcut toward the goal. However, because these five dogs made at least one additional error suggesting an absence of goal tracking, there is little evidence that any of them did actually keep track of the van’s position. E1 and E2, made by a total of seven dogs, also indicated that the dogs failed to keep track of the van’s position. Lastly, E4 (13 dogs) consisted in crossing the street straight ahead, rather than turning left, and E5 and E6 (4 dogs) consisted in continuing straight ahead, rather than moving toward the van, as though the dogs were not keeping track of the van’s position.

No behavioral initiatives were observed, but none were possible.

Median durations for the memorized (207 s) and evaluated (244 s) paths differed, *t(*23) = 14, *p* = 0.00016, and were found to be correlated (ρ = 0.59, *p* = 0.0024).

#### Return/Homing

Ten (43.47%) of the dogs managed to perform this task, while 13 others made a total of 22 errors (see Fig 2). E2 and E3, made by seven dogs, concerned the orientation the dogs had to follow. They suggested that the dogs had not memorized exactly where to turn (i.e. as though they were anticipating a direction to follow (E2) or were trying to take a shortcut (E3). Because five of these seven dogs made further errors, it appears that only two additional dogs kept track of the van’s position, leading to a success rate of 52.17% (12 dogs). D.E. and E1, made by nine dogs in total, did not indicate that the dogs kept track of the van’s position. Finally, E4 (made by six dogs) consisted in crossing the street straight ahead instead of turning right, as though the dogs were not keeping track of the van’s position.

Initiatives were also recorded: four dogs crossed the Avenue des Galavards at the shortest possible location (I1), and six dogs crossed the same avenue a little further along, at a pedestrian crossing (I2), suggesting that the dogs were keeping track of the goal direction.

Median durations for the memorized (311 s) and evaluated (359 s) paths differed, *t(*23) = 11, *p* = 0.00017, and were found to be correlated (*ρ* = 0.61, *p* = 0.0016).

#### Shortcut

Eighteen (78.3%) of the dogs successfully completed the task, and the five other dogs made a total of six errors. The first two errors (D.E. and E1) were made by a single dog, and this dog later performed the shortcut, suggesting that it had been able to recall the goal’s location, albeit rather late in the course of navigation. Three dogs reproduced the memorized path, and one dog made E3 after the shortcut, but before the van was visible, suggesting that it had lost the direction of the goal.

Two of these errors did not concern the shortcutting ability per se, but pertained to the dogs’ reproduction ability, occurring before the shortcutting (see errors marked with an * in Table 3). Their removal led to a corrected success rate of 83% for the 19 dogs and a total of four errors for the four remaining dogs.

No behavioral initiatives were observed, but none were possible.

We based our calculation of the median durations of the memorized and evaluated paths on the dogs that had made no errors either at or after the choice point (i.e. the 19 dogs above). These medians (213 s and 169 s) differed, *t*(19) = 2, *p* = 0.00018, and were found correlated (*ρ* = 0.47, *p* = 0.037).

#### Detour

Sixteen (69.57%) of the dogs successfully completed the task, and the other seven dogs made a total of nine errors. Most of these errors involved continuing along the sidewalk instead of crossing the Avenue Grande Terre on the left (E2 = five dogs), suggesting that the dogs had not memorized exactly where to turn. One dog crossed the avenue long before the pedestrian crossing (E1). This could have been an anticipatory initiative, suggesting that the dog was keeping track of the goal’s direction, but given that all the dogs in the pretests that chose this path became lost, we counted it as an error. One dog failed to avoid the obstacle, continually attempting to pass through it (E3), but once placed in the right direction it managed to perform the detour. There are two possible explanations: either this dog was accustomed to being supported by its owner when confronting obstacles, or it had difficulty inhibiting its behavior when faced with an obstacle and adopting a new rule for an activity). Only two of the dogs that failed the task avoided the obstacle but failed afterwards, although they did manage to complete the path once they had been repositioned (and well oriented) at the spot where the error had taken place (E4 and E5). Interestingly, only three dogs took the northerly detour (cf. S1 Video Detour).

Six of these errors had nothing to do with detouring ability per se, and pertained instead to reproduction ability, as they occurred before the detour (errors marked with an * in Table 3). Excluding these errors led to a corrected success rate of 86.95% (20 dogs) and three errors in total among the remaining three dogs.

We recorded one initiative (I1): three dogs crossed left on the Avenue Grande Terre, 30 m before the pedestrian crossing, and then went to the location of the obstacle.

To calculate the median durations of the memorized and evaluated paths, we used the dogs that did not make any errors at or after the obstacle (the 20 above-mentioned dogs), and excluded the three that made the northerly detour, leaving 17 dogs. For these dogs, the median durations for the memorized and evaluated paths were 129 s and 291 s. These medians differed, *t*(17) = 0, *p* = 0.00029, and were found to be correlated (*ρ* = 0.53, *p* = 0.026).

### 4.2 Relationships between the tasks for number of errors and percentages of dogs that succeeded

We calculated separate sets of correlations for numbers of errors and for the percentages of dogs that succeeded between each pair of tasks, and between the pooled reproduction and homing tasks and the pooled shortcut and detour tasks. These revealed that the homing and detour tasks were positively correlated for errors (*ρ* = 0.53, *p* = 0.008). See S1 Table for all other nonsignificant results (-0.2 ≤ *ρ* ≤ 0.53, 0.11 ≤ *p* ≤ 0.69).

### 4.3 Relationships between dogs that succeeded and individual characteristics

Performances on the return/homing task only correlated with the estimation according to the instructor team of the types of paths usually performed (Yates *χ*^*2*^ = 4.38, Yates *p* = 0.036), and with the degree of visual disability (*χ*^*2*^ = 6.3, *p* = 0.04): the poorer an owner’s vision and the more that owner took regular + many unknown paths, the better his/her dog performed on the homing task. For all other nonsignificant correlations, see S2 Table: -0.017 ≤ *ρ* ≤ 0.25, 0.24 ≤ *p* ≤ 0.93; 0.0007 ≤ Yates *χ*^*2*^ ≤ 1.82, 0.17 ≤ Yates *p* ≤ 0.97; 1.13 ≤ *χ*^*2*^ ≤ 3.1, 0.21 ≤ *p* ≤ 0.56.

Lastly, we analyzed the extent to which the estimation of path types taken by the dyads on a daily basis differed between instructors and owners. The Wilcoxon signed-rank test showed no difference between the two groups, *t(*23) = 2.5, *p* = 0.36, and the two sets of data were correlated (*ρ* = 0.86, *p* < 0.00001).

## Discussion

To the best of our knowledge, the present study is the first to have provided a detailed portrayal of the behaviors of dogs (here, guide dogs) in four distinct tests targeting the four main navigational tasks: *reproducing* a previously travelled path and performing a return trip (*homing*) along a previously travelled path (simple memory of places); and *shortcutting* and *detouring* along a previously travelled route (manipulation of mental representations) [9]. We obtained an overview of the abilities of a group of guide dogs owned by severely impaired persons since years.

First, it is important to remember that the paths had only been followed once. Few dogs were able to reproduce the path (30.43%) or manage the return trip (43.47%) for paths of 300-400 m. Among these dogs who failed, we further were unable to find errors that would suggest they were tracking the location of the goal except for the return task, leading to a success rate of 52.17%. The dogs apparently performed better on the homing task than on the reproduction task, even though the homing path was longer. One possible explanation is that homing is a task for which dogs are explicitly trained (“Find home” instruction), whereas they are not trained to memorize a path per se (i.e. they do it implicitly). This task is commonly performed when exploring new areas from home. Finally, this task is essential for the survival of any animal [8,9]. Thus, guide dogs have developed good homing skills. Interestingly, for both these tasks, we also found that half the dogs made more than one error (8/16 dogs for reproduction, and 6/13 dogs for homing). These descriptive results cannot be compared with those of any previous work, but confirm what instructors have observed, namely, that dogs need to have made many more journeys along such long paths in order to successfully complete wayfinding tasks. According the author SB who is a senior guide instructor, being able to perform without error the reproduction path and homing task may require between three to four trials according to dogs. Shortcutting may require in average two trials thought some dogs may never manage and some may succeed earlier as seen in the present study. Finally, detour may require the same learning trials as those needed in the shortcut task, thought more variability between dogs may be expected. The present results therefore provide a clear indication of the learning rate with one single journey.

Two cognitive processes are engaged in mammal navigation (e.g. [34]. One of the primary sources for navigation is *path integration*. This relies on monitoring self-movement, either dead reckoning on the basis of velocity information, or inertial navigation on the basis of acceleration information [35–38]. Path integration allows to reproduce a travelled distance and perform changes of direction. A second primary source for navigation is *piloting*, or landmark-based navigation: the use of visual environmental details can support or replace the integration of changes in orientation, allowing for places to be identified and providing cues to be used as landmarks where changes in direction occur [39]. This means that routes are easily disrupted if a landmark is removed [36, 39, 40]. Ultimately, when exploring, places become linked by idiothetic (self-movement) inputs [40–43]. These processes have both been shown to take place in dogs, but have never previously been observed in urban settings (for path integration, see [43, 44]; for landmark-based navigation, see [25, 45, 46]. The results for the reproduction and return tasks show that although a small proportion of dogs assimilated all the information during a single journey, and perhaps kept track of the van’s position while navigating, the nature of the errors made by the other dogs suggests that they had only a partial or segmented ability to memorize distances and directions to be followed, as well as landmarks where directions had to be taken. This is line with processes evidenced in humans by [41–43], and with the use of an egocentric frame of reference at the beginning of learning. Furthermore, it is worth noting that the number of errors seemed to increase at the end of the path in the reproduction task, suggesting a recency effect [47, 48]). This is congruent with data showing that path integration errors in humans increase with path length (e.g. [49]). We did not observe this effect for the homing task, suggesting that processing the explored path in reverse order interferes with any recency effect. Finally, some of the errors and initiatives displayed in the homing-but not the reproduction-task by some of the guide dogs indicate that these dogs were keeping track of the goal’s location, as they behaved as though attempting to shortcut the path.

The dogs performed the shortcut task on a shorter path than the two previous ones, and more than 80% of them successfully completed it. In an open field, [50] led dogs from a starting point to a location with food hidden behind bushes. The dogs were then walked back to the starting point. Next, they were led to a second location with food hidden behind bushes, then led back to the starting point. In 96% of the trials, the dogs first ran to the closest food location and then ran directly to the second hidden food location. The dogs therefore integrated the whereabouts of the second food location in relation to the first, although it is impossible to eliminate the use of distal, or even subtle, proximal landmarks indicating the direction of the second goal. The present result converges with that of [50] and indicates that a proportion of the dogs performed successfully in a larger urban area where no distal landmarks could be used. Their success rate suggests that with a shorter path than the two previous ones, the guide dogs were able to keep track of the goal and to compute the shortest path from the start, which is congruent with results in humans [49], though not in rats [51]. It should be noted that this path involved reproducing a very short portion of path (the beginning). Once again, there were few errors, suggesting that path length (e.g. [50]) affects the ability to reproduce it.

The detour task per se was successfully performed by 86.95% of the dogs. This ability has already been extensively described in various species [52] and in domestic dogs or dingoes in small-scale laboratory settings, using V-shaped barriers (for a review, see [53]). Furthermore, [29] found evidence that dogs code the position of a target according to the different objects around it, and use dead reckoning in a small-scale setting, with the two encoding modes being employed differently, according to the complexity of the detour. The present task showed how well the guide dogs were able to perform a detour in a large-scale urban environment after a single journey, based on path integration information. Interestingly, most dogs detoured to the south (20/23). The simplest explanation for this southerly detour is that the dogs turned left at the obstacle, confirming an egocentric strategy. Finally, a longer portion had to be reproduced for the detour than for the shortcut task, but it was still shorter than that of the reproduction task. The number of errors was again congruent with the length of the path. Furthermore, although the dogs had travelled in a closed van, we cannot fully dismiss the possibility that the dogs performed path integration [24], which would partially account for their performances. The fact that most of the dogs could shortcut and detour (and for the detour task, they could cross before the pedestrian crossing they had taken earlier) also highlights and confirms the dogs’ ability to adjust to the task with flexibility, taking suitable initiatives for navigating toward the previously memorized goal (see also initiatives for the return/homing task).

Data were compatible with the use of an egocentric encoding strategy that is reliable for short paths but not for longer ones after a single journey of incidental learning, suggesting segmented integration of the path’s characteristics. It should be pointed out that these behaviors were obtained with verbal encouragements and food rewards, matching the usual conditions in which guide dogs are taught new paths.

We further found that the durations of the memorized and evaluated paths were positively correlated for each task, suggesting similar processes across tasks. Moreover, the fact that the duration of the evaluation phase exceeded that of the memorization phase for the reproduction and return tasks suggests that the dogs engaged in different activities in these two phases: whereas they mainly followed instructions during the first phase, with a walk facilitated by the sighted handler, they required time for spatial processing during the evaluation phase.

Our hypothesis concerning spatial memory abilities, which predicted links between path reproduction and homing (simple memory of places) and between shortcutting and detouring (manipulation of mental representations, 52) was not confirmed. In other words, we did not find that the cognitive processing of the two routine tasks were associated neither that the two tasks requiring plasticity of representations were associated, with one learning trial. We did, however, find a correlation between errors (though not performances that are more rough -i.e. success vs. failure) on homing and detouring, likely because these two vital tasks are undertaken on a daily basis, suggesting an effect of experience. This is in accordance with the performances found on homing, a crucial survival ability, that were better when a) the dog’s owner had a particularly severe visual disability, probably because there was no navigational interference with the owner’s remaining sight, and b) the dog regularly experienced new journeys, suggesting an effect of wayfinding experiences. Finally, neither the age of the dogs (*M* = 5.4 years, *SE* = 1.57) nor the amount of time they had been with their owner (*M* = 2.65, *SE* = 1.51) accounted for their wayfinding abilities, probably because the group was quite homogeneous for these variables.

Additionally to providing qualitative and quantitative knowledge on wayfinding abilities in the dog species, this study and this protocol purvey a tool or at least a foundation to improve various aspects of dogs teaching and matching the guide dogs with blind persons. For instance, guide dogs from various schools could be tested to compare the effects of learning methods, that can be based on more or less positive methods. Moreover, the scores of the dogs, after one or several learning trials, could help to match better guide dogs and blind persons profiles; for instance a person used to navigate very little and to perform always the same paths should get a guide dog that is good at reproduction and homing tasks rather than at the two other tasks, whereas a person who navigate a lot and in unfamiliar urban areas may need all the wayfinding abilities. Knowing how many trials are required per dog to succeed each task will allow to determine its learning rate and thus to facilitate the matching of the dogs with the abilities and needs of the blind persons.

## Conclusion

In the present study, we developed and applied an elaborate protocol for systematically studying four wayfinding abilities in guide dogs, applying a natural way of motivating them. We evidenced the number and type of errors and initiatives made by guide dogs after a single journey highlighting the cognitive processes at play, and two characteristics of the dyads that affected performances. These four tasks enabled us to establish the dogs’ profile in the present experimental conditions: they showed that they were not good at memorizing long paths after a single journey, but were good at shortcutting and detouring for short paths. Moreover, their homing ability was better when they were used to travelling in new areas, showing an effect of earlier navigational experiences, and when their owners had very little residual sight. In future studies dogs should also be tested after more than one journey along the paths, in order to gain further insight into how dogs encode urban environments over time. The effect of the magnetic field on these abilities could also be assessed.

## Supporting information

S1 Table Results of the Spearman correlations for the relationships between wayfinding tasks on numbers of errors and percentages of dogs that succeeded (percentages corrected for shortcut and detour tasks to 83% and 86.95%) (*N* = 23). (DOCX)

S2 Table Results of the Spearman correlations and chi-square analyses (Yates’s corrections) for the relationship between the percentages of dogs that succeeded (percentages corrected for the shortcut and detour tasks to 83% and 86.95%) (*N* = 23) and their individual characteristics (Regular and Regular + few unknown vs. Regular + many unknown). (DOCX)

S1 Text In the present guide dog school, when a guide dog is given to an owner, the dog navigates in the owner’s neighborhood a number of times with its owner, along the paths the owner will have to take daily, with the help of the dog’s trainer. This way, the dog is exposed to its new owner’s verbal instructions, and learns the paths between (named) locations. It thus becomes familiar with new paths and named locations.
(DOCX)

S2 Text As the behaviors recorded in the videos were unambiguous and easily identifiable (i.e. wrong directions taken), and no hypotheses had been formulated, we did not foresee any bias on the judges’ part. (DOCX)

S1 Video Detour. This video present one guide dog performing the training phase and the wayfinding phase for the Detour task.
(WMV)
