# Supplementary material for: Guide dogs' navigation after a single journey: A descriptive study of path reproduction, homing, shortcut and detour
Source: PLoS One. 2019 Jul 16;14(7):e0219816. doi: 10.1371/journal.pone.0219816 (PMC6634399; doi:10.1371/journal.pone.0219816)
Supplement: S1 Table — (DOCX) [file pone.0219816.s001.docx]

| **Correlation** | **Errors** | **Performances** |
| --- | --- | --- |
| **Reproduction vs. Homing** | *ρ* = 0.15  *p* = 0.49 | *ρ* = 0.18  *p* = 0.405 |
| **Reproduction vs. Shortcut** | *ρ* = -0.2  *p* = 0.36 | *ρ* = 0.18  *p* = 0.405 |
| **Reproduction vs. Detour** | *ρ* = 0.13  *p* = 0.55 | *ρ* = -0.19  *p* = 0.372 |
| **Homing vs. Shortcut** | *ρ* = 0.25  *p* = 0.24 | *ρ* = 0.17  *p* = 0.43 |
| **Homing vs. Detour** | *ρ* = 0.53  ***p* = 0.008** | *ρ* = 0.34  *p* = 0.11 |
| **Shortcut vs. Detour** | *ρ* = 0.16  *p* = 0.46 | *ρ* = 0.16  *p* = 0.46 |
| **Reproduction + Homing vs. Shortcut + Detour** | *ρ* = 0.21  *p* = 0.33 | *ρ* = 0.09  *p* = 0.69 |
