# Supplementary material for: Guide dogs' navigation after a single journey: A descriptive study of path reproduction, homing, shortcut and detour
Source: PLoS One. 2019 Jul 16;14(7):e0219816. doi: 10.1371/journal.pone.0219816 (PMC6634399; doi:10.1371/journal.pone.0219816)
Supplement: S2 Table — (DOCX) [file pone.0219816.s002.docx]

S2_Table. Results of the Spearman correlations and chi-square analyses (Yates’s corrections) for the relationship between the percentages of dogs that succeeded (percentages corrected for the shortcut and detour tasks to 83% and 86.95%) (*N* = 23) and their individual characteristics (Regular and Regular + few unknown vs. Regular + many unknown).

|  | **Age**  **(in years)** | **Time with owner**  **(in years)** | **Sex** | **Degree of owner’s visual disability** | | **Types of paths usually taken** | | |  |
| --- | --- | --- | --- | --- | --- | --- | --- | --- | --- |
|  |  |  |  |  |  | **According to instructor team** | **According to owner** | |  |
| **Reproduction** | *ρ* = -0.17  *p* = 0.43 | *ρ* = -0.06  *p* = 0.77 | Yates χ^2^ = 0.019  Yates *p* = 0.89 | | χ^2^ = 1.47  *p* = 0.47 | Yates χ^2^ = 0.001  Yates *p* = 0.97 | | Yates χ^2^ = 0.11  Yates *p* =0.73 | |
| **Homing** | *ρ* = -0.1  *p* = 0.63 | *ρ* = -0.09  *p* = 0.67 | Yates χ^2^ = 0.057  Yates *p* = 0.81 | | χ^2^ = 6.3  ***p* = 0.04** | Yates χ^2^ = 4.38  Yates *p* = **0.036** | | Yates χ^2^ = 1.82  Yates *p* = 0.17 | |
| **Shortcut** | *ρ* = 0.06  *p* = 0.75 | *ρ* = -0.017  *p* = 0.93 | Yates χ^2^ = 0.2  Yates *p* = 0.64 | | χ^2^ = 1.13  *p* = 0.56 | Yates χ^2^ = 0.24  Yates *p* = 0.62 | | Yates χ^2^ = 0.24  Yates *p* = 0.62 | |
| **Detour** | *ρ* = 0.25  *p* = 0.24 | *ρ*= 0.25  *p* = 0.24 | Yates χ^2^ = 0.0007  Yates *p* = 0.93 | | χ^2^ = 3.1  *p* = 0.21 | Yates χ^2^ = 0.52  Yates *p* = 0.81 | | Yates χ^2^ = 0.52  Yates *p* = 0.81 | |
