# Supplementary material for: Guide dogs' navigation after a single journey: A descriptive study of path reproduction, homing, shortcut and detour
Source: PLoS One. 2019 Jul 16;14(7):e0219816. doi: 10.1371/journal.pone.0219816 (PMC6634399; doi:10.1371/journal.pone.0219816)
Supplement: S1 Text — This way, the dog is exposed to its new owner’s verbal instructions, and learns the paths between (named) locations. It thus becomes familiar with new paths and named locations. (DOCX) [file pone.0219816.s003.docx]

S1_Text

In the present guide dog school, when a guide dog is given to an owner, the dog navigates in the owner’s neighborhood a number of times with its owner, along the paths the owner will have to take daily, with the help of the dog’s trainer. This way, the dog is exposed to its new owner’s verbal instructions, and learns the paths between (named) locations. It thus becomes familiar with new paths and named locations.
