# Supplementary material for: Guide dogs' navigation after a single journey: A descriptive study of path reproduction, homing, shortcut and detour
Source: PLoS One. 2019 Jul 16;14(7):e0219816. doi: 10.1371/journal.pone.0219816 (PMC6634399; doi:10.1371/journal.pone.0219816)
Supplement: S2 Text — (DOCX) [file pone.0219816.s004.docx]

S2_Text

As the behaviors recorded in the videos were unambiguous and easily identifiable (i.e. wrong directions taken), and no hypotheses had been formulated, we did not foresee any bias on the judges’ part.
